# Sea buckthorn polyphenols improve lipid metabolism via gut–liver axis modulation in db/db mice

**DOI:** 10.29219/fnr.v70.13777

**Published:** 2026-07-31

**Authors:** Zongzhen Guo, Ziyi Yuan, Tiangang Xu, Yian Yao, Yiyun Zhang, Zongxin Shao, Qun Shen, Yong Xue

**Affiliations:** 1 National Engineering and Technology Research Center for Fruits and Vegetables, College of Food Science and Nutritional Engineering, China Agricultural University, Beijing, P.R. China; 2 Research Centre of Clinical Epidemiology, Peking University Third Hospital, Beijing, China; 3 National Center of Technology Innovation (Deep Processing of Highland Barley) in Food Industry, China Agricultural University, Haidian District, Beijing, P.R. China

**Keywords:** sea buckthorn polyphenols, *db/db* mice, lipid metabolism, gut microbiota, cholesterol homeostasis

## Abstract

Sea buckthorn polyphenol extract (SPE) has been reported to exert beneficial effects on lipid metabolism, yet its bioactive constituents and underlying mechanisms remain insufficiently defined. In this study, SPE was administered to *db/db* mice to evaluate its impact on lipid metabolism and gut microbiota, and key polyphenols were further investigated in HepG2 cells combined with a network pharmacology approach. In *db/db* mice, SPE restored serum high-density lipoprotein (HDL) levels and alleviated hepatic steatosis, accompanied by a tendency toward reduced body weight gain. These changes were accompanied by marked improvements in gut dysbiosis, with increased relative abundances of beneficial genera such as *Lactobacillus* and *Akkermansia*. High-performance liquid chromatography (HPLC) analysis identified isorhamnetin, myricetin, kaempferol, quercetin, and rutin as the major polyphenols in SPE, and all of these constituents decreased intracellular cholesterol and triglyceride accumulation in HepG2 cells without obvious cytotoxicity. Network pharmacology analysis focusing on isorhamnetin and myricetin revealed overlapping targets with obesity-, nonalcoholic fatty liver disease-, and diabetes-related genes that were mainly enriched in lipid-metabolism–related processes and pathways, including fatty acid metabolism, cholesterol homeostasis, nonalcoholic fatty liver disease, lipid and atherosclerosis, and PPAR/AMPK signaling. Integration of gut microbiota, fecal metabolite, and target information further suggested a gut microbiota–metabolite–host target axis involving beneficial taxa such as *Akkermansia* and *Enterococcus* and central hubs including PPARG, TNF, and IL6. Overall, these findings indicate that SPE improves lipid metabolism through coordinated modulation of gut microbiota and hepatic cholesterol–lipid homeostasis, supporting its potential use in the dietary management of obesity and dyslipidemia.

## Popular scientific summary

- This study shows that sea buckthorn polyphenol extract (SPE) improves obesity-related blood lipid disorders in a genetic obesity mouse model by reshaping gut microbiota and normalizing liver cholesterol metabolism.
- Combining animal experiments, cell studies, and network analysis, we highlight key polyphenols and microbes linked to better lipid control.
- These findings support SPE as a promising dietary strategy or functional food ingredient for managing metabolic disorders.

Obesity, affecting over one billion individuals worldwide, is closely linked to lipid metabolism disorders (1), characterized by elevated triglycerides (TG), total cholesterol (TC), low-density lipoprotein cholesterol (LDL-C), reduced high-density lipoprotein cholesterol (HDL-C), hepatic steatosis, insulin resistance, and chronic low-grade inflammation (2–5). Dysregulated lipid metabolism contributes to metabolic syndromes including type 2 diabetes, atherosclerosis, and nonalcoholic fatty liver disease (NAFLD). Dietary modulation of lipid metabolism is a safe strategy that offers cardio-metabolic benefits beyond pharmacotherapy (6, 7). Exploiting the therapeutic potential of drugs and medicinal foods for modulating obesity and lipid metabolism is a critical pathway toward alleviating related metabolic disorders.

Sea Buckthorn (*Hippophae rhamnoides Linn.*) is a shrub renowned for its medicinal and edible properties (8). It is rich in various bioactive compounds, particularly polyphenols, carotenoids, and unsaturated fatty acids (9, 10). The polyphenol extract from sea buckthorn berries, termed Sea Buckthorn Berry Polyphenol Extract (SPE), is enriched with potent bioactive components including phenolic acids (such as chlorogenic and caffeic acid), flavonoids (such as isoquercetin, quercetin, and their glycosides), and flavanols (11). Polyphenols are known to reduce lipid accumulation via activation of AMPK/SIRT1 pathways, promoting lipolysis and mitochondrial biogenesis (12). Recent studies have shown that the polyphenol extract from sea buckthorn berries (SPE) exhibits antioxidant, anti-inflammatory, immunoregulatory, and metabolic enhancement properties (13, 14).

Gut microbiota, recognized as a ‘hidden organ’ of the human body, plays a central role in host energy balance, nutrient metabolism, and immune regulation (15). The in vitro experiments showed that sea buckthorn was a rich source of prebiotic substrates which could significantly stimulate the growth of beneficial intestinal microbiota. The enrichment of *Lactobacillus* and *Akkermansia* genera highlighted the prebiotic activity of SPE. *Akkermansia muciniphila* can degrade mucin to produce short-chain fatty acids (SCFAs) and strengthen the epithelial barrier function (16). *Lactobacillus spp.* can regulate bile acid metabolism through the activity of bile salt hydrolase to affect the solubility and excretion of cholesterol (17). SCFAs (such as acetate, propionate) may activate gluconeogenesis in the intestine or AMPK pathway in the liver (18), thus synergistically contributing to the increase of HDL-C and decrease of LDL-C. The microbiota–liver axis is one of the important pathways for the systemic effects of SPE.

In parallel, network pharmacology has emerged as a powerful systems-level strategy for elucidating the multicomponent and multitarget mechanisms of natural products and functional foods. By integrating compound profiling with target prediction, disease-associated genes, and pathway enrichment, network pharmacology can reveal how complex mixtures such as polyphenol extracts act on interconnected signaling networks rather than single targets (19, 20). When combined with gut microbiota and metabolomics data, this approach further enables the construction of bacteria–metabolite–host target networks, providing mechanistic insight into how specific microbial taxa and their metabolites participate in host lipid and cholesterol homeostasis (21). Thus, network pharmacology is particularly suitable for dissecting the integrated actions of SPE-derived polyphenols on obesity-related metabolic pathways.

Despite these findings, the systemic effects of SPE on lipid metabolism in genetically obese *db/db* mice, and the underlying mechanisms involving gut microbiota and hepatic cholesterol pathways remain unclear. Therefore, this study aims to elucidate the lipid metabolism mechanisms of SPE in *db/db* mice by integrating in vivo experiments, in vitro validation, and network pharmacology analysis. The primary polyphenols in SPE will be ensured using high-performance liquid chromatography (HPLC) analysis. In vitro evidence will confirm the molecular mechanisms of SPE involved in cholesterol metabolism, thus revealing the molecular basis for its potential to ameliorate dyslipidemia and restore cholesterol homeostasis. We hypothesize that SPE alleviates dyslipidemia by modulating the gut microbiota and regulating hepatic cholesterol metabolism pathways. The findings will provide experimental evidence to support the development of SPE as a functional food or natural remedy for obesity-related metabolic disorders.

## Materials and methods

### Materials and reagents

SPE was purchased from Ningxia Hengruikang Biotechnology Co., Ltd. (Ningxia, China). Low-fat diet (LFD) (D12450J, 10 kcal% fat) was obtained from Beijing Yicheng Science and Technology Co., Ltd. (Beijing, China). Phosphate-buffered saline (0.1 M), paraformaldehyde, and 75% ethanol were obtained from Solarbio (Beijing, China). TG, TC, HDL-C, and LDL-C assay kits were purchased from Nanjing Jiancheng Bioengineering Institute (Nanjing, China). Dulbecco’s Modified Eagle Medium (DMEM) and fetal bovine serum (FBS) were obtained from the National Experimental Cell Resource Sharing Service Platform (Beijing, China). Total RNA extraction kit and PrimeScript™ RT reagent kit were purchased from Tiangen Biotech (Beijing, China) and Takara Bio Inc. (Kusatsu, Japan), respectively. Other chemical reagents, including quercetin, were obtained from Macklin Biochemical Technology Co., Ltd. (Shanghai, China).

### Animal experiments

Six male C57BL/6J mice (20 ± 2 g) and 12 male *db/db* mice (36 ± 2 g), 6 weeks old, were purchased from Changzhou Cavens Experimental Animal Co., Ltd. Mice were housed in SPF conditions (23 ± 2°C, 55 ± 5% humidity, 12-h light/dark) with ad libitum access to water and LFD (D12450J, Beijing Yicheng Science and Technology Co., Ltd.) for 1 week of acclimatization. C57BL/6J mice served as the control group (Control, *n* = 6) and were maintained on one LFD. *Db/db* mice were randomly assigned to a diabetic model group (Model, *n* = 6) or an intervention group (Inter, *n* = 6), both fed the same LFD. Inter-group received daily oral gavage of SPE (300 mg/kg body weight) (14) for 8 weeks, while Control and Model groups received equivalent volumes of distilled water. SPE, stored at −80°C protected from light, was freshly dissolved and adjusted weekly to maintain a gavage volume of 0.2–0.4 mL.

Body weight and food intake were measured weekly. After 8 weeks, mice were anesthetized and euthanized. The blood samples for biochemical analysis were obtained from mice after they were sacrificed following a 12-h overnight fast and isolated at 4°C (4,000 rpm, 10 min) to obtain serum. In addition, liver, cecal contents, colon segments, and epididymal adipose tissue were collected and stored at −80°C. All procedures were approved by the Experimental Animal Welfare and Ethics Committee of University (Approval No. AW08012020-4).

### Histological analysis

Histological examination of each tissue type was conducted following established protocols from a previous study (22). Briefly, tissues were fixed in 4% paraformaldehyde (v/v), dehydrated through a graded ethanol series, and embedded in paraffin. Sections of 4-μm thickness were cut using a microtome (RM2016, Shanghai Leica Instruments Ltd, Shanghai, China) and air-dried. After dewaxing, the sections were stained with hematoxylin and eosin (H&E), mounted with neutral gum, and dried prior to microscopic observation. For liver tissues, Oil Red O staining was additionally performed. The tissues were dehydrated, dried, embedded, and sectioned at 8–10 μm thickness using a freezing microtome (Thermo, Massachusetts, USA). Following staining with Oil Red O, the sections were sealed and examined under a microscope (BX51, Olympus Corporation, Tokyo, Japan).

### Cell culture and treatment

HepG2 cells were obtained from the National Experimental Cell Resource Sharing Service Platform and cultured in DMEM supplemented with 10% FBS and 1% penicillin-streptomycin at 37°C with 5% CO_2_. Cells were subcultured at 80–90% confluency using 0.25% trypsin-EDTA. Cryopreserved cells were thawed and recovered in complete medium, with long-term storage in liquid nitrogen. The effect of sea buckthorn monophenols on cell viability was assessed using the 3-(4,5-dimethylthiazol-2-yl)-2,5-diphenyltetrazolium bromide (MTT) assay.

### Biochemical parameters

TC, TG, LDL-C, and HDL-C were measured using commercial kits following manufacturer instructions (Nanjing Jiancheng Institute of Bioengineering, Nanjing, China).

### Gut microbiota analysis

Bacterial genomic DNA was extracted from fecal samples using the E.Z.N.A.® Soil DNA Kit (Omega Bio-tek, Norcross, GA, USA). DNA purity and concentration were assessed with 1% agarose gel electrophoresis and a NanoDrop 2000 spectrophotometer (Thermo Fisher Scientific, Waltham, MA, USA). The V3–V4 region of the 16S rRNA gene was amplified using primers 338F (5′-ACTCCTACGGGAGGCAGCAG-3′) and 806R (5′-GGACTACHVGGGTWTCTAAT-3′) on an ABI GeneAmp® 9700 thermocycler. PCR products were separated on 2% agarose gels, purified using the AxyPrep DNA Gel Extraction Kit (Axygen Biosciences, Union City, CA, USA), and quantified with a Quantus™ Fluorometer (Promega, Madison, WI, USA). Libraries were constructed using the NEXTflex™ Rapid DNA-Seq Kit (Bioo Scientific, Austin, TX, USA) and sequenced on the Illumina MiSeq PE300 platform. Raw sequences were quality controlled using fastp (v0.20.0) and merged with FLASH (v1.2.7). OTUs were clustered at 97% similarity using UPARSE (v7.1) with chimera removal. Taxonomic classification was performed with the RDP classifier (v2.2) against the SILVA 16S rRNA database (v138) at a 70% confidence threshold.

### Qualitative analysis of polyphenols in sea buckthorn

SPE composition was analyzed by HPLC to quantify major polyphenols (23). Standard stock solutions of the seven compounds were meticulously prepared in methanol. Each solution was subsequently filtered through a 0.45-μm membrane filter before direct injection. The chromatographic conditions utilized included an Agilent ZORBAX SB-C18 column (4.6 mm × 250 mm, 5 μm) on an Agilent 1200 system, equipped with DAD for chromatography. The detection wavelength was consistently set at 280 nm. A flow rate of 1.0 mL/min and an injection volume of 10 μL were maintained throughout the process. All chromatographic procedures were conducted at ambient temperature. The mobile phase consisted of methanol (B), containing 0.4% phosphoric acid (A)-water. The gradient elution sequence was as follows: 0–10 min, 80–70% A; 10–20 min, 70–60% A; 20–30 min, 60–50% A; 30–40 min, 50–40% A; 40–45 min, 40–30% A. Prior to use, the mobile phases were filtered through a 0.45-μm membrane filter and then sonicated. The retention times of 7 standards including protocatechuic acid, isorhamnetin, caffeic acid, kaempferol, myricetin, quercetin, and rutin were shown in S1.

### Collection of compound-related targets

The chemical information of myricetin and isorhamnetin, including the canonical SMILES strings and 2D structures, was obtained from PubChem (https://pubchem.ncbi.nlm.nih.gov). Potential human targets of each flavonoid were predicted using three web-based platforms: SwissTargetPrediction (http://www.swisstargetprediction.ch, input: canonical SMILES), PharmMapper (https://www.lilab-ecust.cn/pharmmapper/index.html, input: 2D structure file), and the Comparative Toxicogenomics Database (CTD) (https://ctdbase.org/, input: compound name). The predicted targets from the three platforms were merged, converted to official human gene symbols according to UniProt, and duplicates were removed to obtain the final sets of putative protein targets for myricetin and isorhamnetin.

### Collection of disease-related targets

Disease-related genes were collected from the GeneCards database (https://www.genecards.org). The keywords ‘obesity’, ‘NAFLD’, and ‘diabetes’ were used to retrieve genes associated with each condition. For subsequent analysis, only genes with a relevance score ≥ 20 were retained. For each disease, all entries were mapped to standardized human gene symbols using UniProt, and redundant records were removed to generate the final obesity-, NAFLD- and diabetes-related target sets.

### Identification of common targets and construction of PPI networks

For each compound–disease pair (myricetin–obesity, myricetin–NAFLD, myricetin–diabetes, isorhamnetin–obesity, isorhamnetin–NAFLD, and isorhamnetin–diabetes), the intersection between the corresponding compound-related targets and disease-related targets was calculated to define the common targets. These overlaps were visualized as Venn diagrams using an online bioinformatics platform (http://www.bioinformatics.com.cn).

All intersecting targets for each compound–disease pair were then imported into the STRING database (https://string-db.org, version 12.0; organism set to Homo sapiens) to construct protein–protein interaction (PPI) networks. The interaction data were exported and visualized in Cytoscape (version 3.9.1). In the resulting networks, node size and color were mapped to the degree value (bigger and darker nodes indicate higher degree value), and edge thickness and color both reflected the combined interaction score (thicker and darker edges indicate higher combined scores).

### GO and KEGG enrichment analysis and visualization

To functionally characterize the common targets, Gene Ontology (GO) and Kyoto Encyclopedia of Genes and Genomes (KEGG) enrichment analyses were performed using the DAVID database (https://davidbioinformatics.nih.gov/), with Homo sapiens specified as the background. For each compound–disease gene set, significantly enriched GO terms (classified into biological process, cellular component, and molecular function) and KEGG pathways were identified based on *P*-values. GO results were ranked in ascending order of *P*-value, and the top 10 terms in each GO category (or all terms if fewer than 10 were significant) were selected. For KEGG analysis, the top 20 pathways with the lowest *P*-values were retained.

The selected GO terms and KEGG pathways were visualized as bubble plots using the Microbiome/Bioinformatics online tool (http://www.bioinformatics.com.cn). In these plots, the x-axis represents fold enrichment, bubble size corresponds to the number of associated genes, and the color gradient reflects − log10 (*P*-value).

### Gut microbiota–metabolite–target network analysis

To explore the potential involvement of the gut microbiota and their metabolites in mediating the effects of myricetin and isorhamnetin, integrated drug–host target–microbiota–metabolite–target networks were constructed by database mining. The drug–disease common targets identified in the network pharmacology analysis were mapped to the host–gene associations in the Gut Microbe–Gene (gutMGene) database (https://bio-computing.hrbmu.edu.cn/gutmgene/) to identify human-relevant gut microbes sharing at least one host gene with each compound–disease set. For each microbe, the number of shared genes was calculated and used to rank microbes; those with the highest counts were defined as core bacteria. Microbe–metabolite associations for these core bacteria were then extracted from gutMGene, and PubChem CIDs were used to obtain SMILES structures of the corresponding metabolites from PubChem (https://pubchem.ncbi.nlm.nih.gov). The SMILES strings were submitted to the Similarity Ensemble Approach (SEA; https://sea.bkslab.org/) (and TargetNet when necessary) to predict putative human protein targets of the microbial metabolites. Finally, drugs, shared host targets, core gut microbes, microbial metabolites, and metabolite-derived host targets were imported into Cytoscape (version 3.9.1) to visualize the integrated bacteria–metabolite–target networks, with node types distinguished by color and edges representing microbe–gene, microbe–metabolite and metabolite–target associations derived from gutMGene and target-prediction platforms.

### Data analysis

Data analysis was performed using SPSS 19.0 (SPSS Inc., Chicago, IL, USA) and R software (version 3.3.1). Graphs were generated with GraphPad Prism 9.0 (La Jolla, CA, USA) and R software. All results are presented as mean ± SEM or median (interquartile range), and differences were considered statistically significant at *P* < 0.05. Statistical comparisons among groups were conducted using one-way analysis of variance (ANOVA) followed by Duncan’s post hoc test, analysis of similarities (ANOSIM), Adonis analysis, Kruskal–Wallis test, or Wilcoxon rank-sum test, depending on the data distribution. Principal coordinate analysis (PCoA) and nonmetric multidimensional scaling (NMDS) based on Bray–Curtis distances at the genus level were applied to evaluate beta diversity. The characteristic bacterial in different groups were identified by linear discriminant analysis (LDA) effect size (LEfSe) analysis (LDA score ≥ 3.0). The Spearman correlation analysis was used to assess relationships between physiological and biochemical parameters and the relative abundance of gut microbiota at the phylum level.

## Results

### SPE reduces body weight and fat mass in db/db mice

**Fig. 1:**
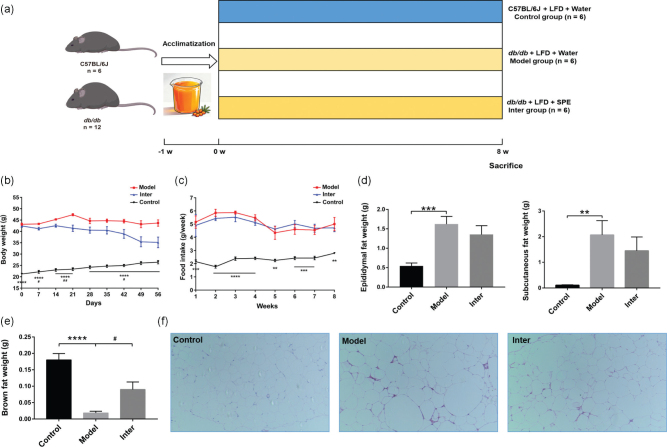
Effect of SPE on body weight, food intake, and adipose tissue in *db/db* mice. (a) Experimental design of the mice model. (b) Body weight changes curve. (c) Food intake changes curve. (d) Subcutaneous and epididymal fat mass. (e) Brown fat mass. (f) H&E staining of epididymal adipose tissue. Comparison of the Model group with the Control group is marked as *, and the comparison of the Model group with the inter-group is marked as #. * *P* < 0.05, ** *P* < 0.01, *** *P* < 0.001, **** *P* < 0.0001, # *P* < 0.05, ## *P* < 0.01. Data are presented as mean ± SEM (*n* = 6 mice per group).

SPE intervention tended to reduce body weight and epididymal/subcutaneous fat mass compared with *db/db* Model mice, although these differences did not reach statistical significance, whereas brown adipose tissue mass was significantly increased in the SPE group. H&E staining showed smaller, more uniform adipocytes in the SPE intervention group (Fig. 1).

As shown in Fig. 1a, the experimental design using *db/db* mice included an 8-week intervention period. The Model group maintained a significantly higher body weight throughout the intervention time compared to Control group, indicating the obesity model was established successfully. When treated with SPE, the body weight significantly reduced (Fig. 1b), while there was no significant difference in food intake between the Inter group and the Model group (Fig. 1c). SPE treatment significantly attenuated the accumulation of both subcutaneous (Fig. 1d) and epididymal fat (Fig. 1f) and promoted a notable increase in brown adipose tissue mass (*P* < 0.05, Fig. 1e). Histological analysis of epididymal fat revealed that Inter-group exhibited smaller and more uniform adipocytes compared to the Model group (Fig. 1f). These morphological and tissue-mass changes demonstrate that SPE ameliorates obesity-related adipose tissue remodeling and promotes a metabolically favorable fat distribution.

### SPE improves serum lipid profile and hepatic steatosis

**Fig. 2:**
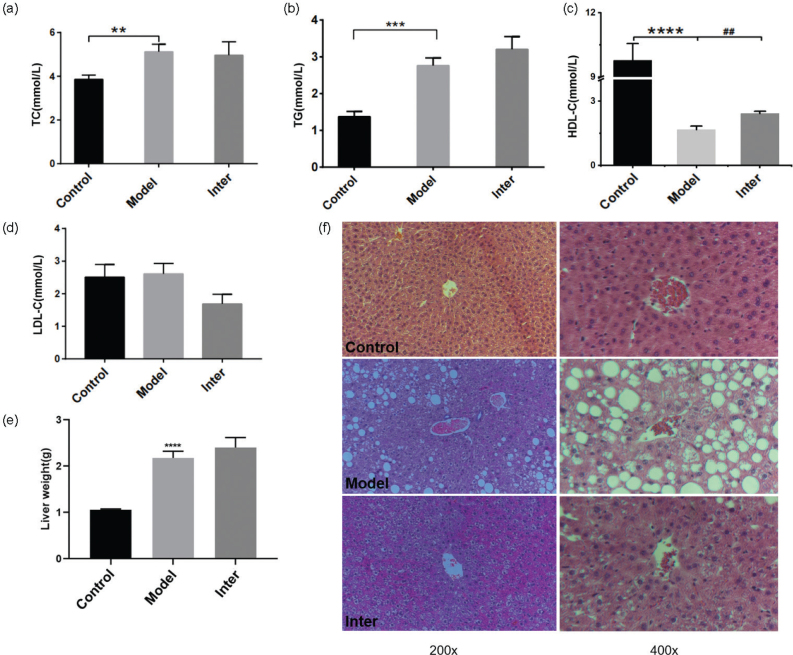
SPE effects on serum lipid profiles and liver histology. (a–d) Serum TG, TC, HDL-C, and LDL-C. (e) Liver weight. (f) H&E staining of liver. Comparison of the Model group with the Control group is marked as *, and the comparison of the Model group with the Inter-group is marked as #. ** *P* < 0.01, *** *P* < 0.001, **** *P* < 0.0001, # *P* < 0.05, ## *P* < 0.01. Data are presented as mean ± SEM (*n* = 6 mice per group).

SPE significantly increased HDL-C levels compared with the Model group (*P* < 0.05), while LDL-C showed a modest, nonsignificant decreasing trend in the SPE group. Liver histology analysis indicated SPE treatment significantly reduced steatosis, inflammatory infiltration, and lipid vacuoles, compared with the Model group (Fig. 2).

Specifically, as shown in Fig. 2, serum levels of TC (Fig. 2a), TG (Fig. 2b), and HDL-C (Fig. 2c) differed significantly between the Model group and Control group (*P* < 0.05), confirming the successful establishment of the obesity model. HDL-C was markedly recovered after SPE treatment (*P* < 0.05). Although no significant differences were observed in LDL-C (Fig. 2d), the trend of its changes were in line with expectations, which increased in the Model group and decreased in the Inter-group.

Liver weight in the model group was significantly greater than in controls (*P* < 0.05). Unfavorably, no difference was found in the Inter-group (Fig. 2e). Histological analysis (Fig. 2f) revealed severe hepatic steatosis, structural disorganization, and inflammatory infiltration in *db/db* Model mice. Interestingly, SPE treatment notably attenuated these pathological changes, resulting in clearer lobular architecture, reduced lipid vacuoles, decreased inflammation, and better-preserved central vein and sinusoid structures. The changes in the SPE-treated group showed the internal structure of the liver tended to resemble that of the negative control group. These results indicated that SPE could improve serum lipid profiles and alleviate hepatic steatosis and tissue injury in *db/db* mice, supporting its beneficial role in modulating lipid metabolism and reducing liver fat accumulation.

### SPE modulates gut microbiota composition

**Fig. 3:**
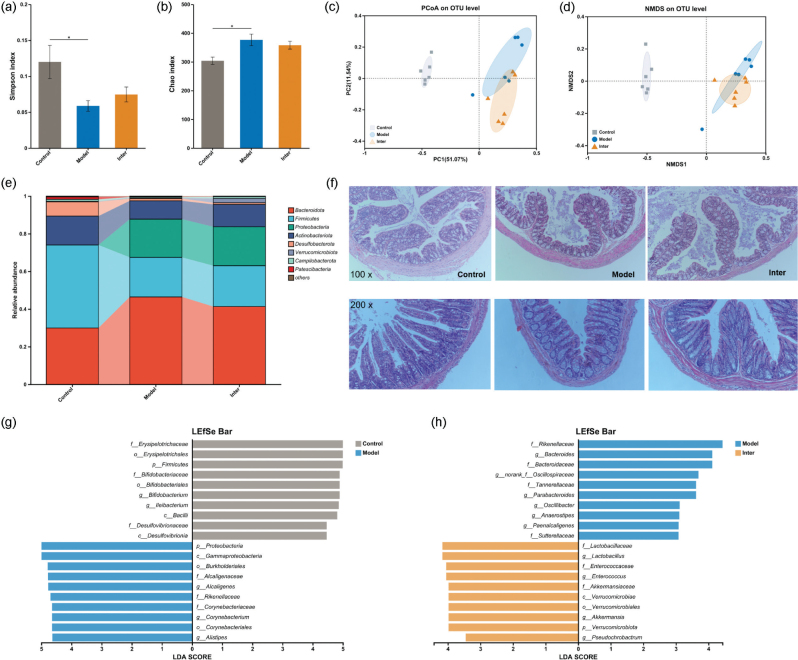
SPE effects on gut microbiota composition and colon histopathology. (a) Simpson diversity index across different groups. (b) Chao1 index for estimating microbial richness. (c) Principal coordinates analysis (PCoA) based on OTU-level profiles. (d) nonmetric multidimensional scaling (NMDS) score based on OTU-level profiles. (e) Relative abundance of gut microbiota at the phylum level. (f) Representative H&E-stained sections of colon tissue. (g, h) Analysis results of LEfSe in the gut microbiota of mice in different groups (LDA value distribution map). Comparison of the Model group with the Inter-group is marked as *, * *P* < 0.05, ** *P* < 0.01. Data are presented as mean ± SEM (*n* = 6 mice per group).

SPE intervention helped restore gut microbiota homeostasis by modulating the relative abundances of dominant phyla and enriching beneficial genera, including a decrease in Bacteroidetes and an increase in Actinobacteria, alongside higher abundances of *Akkermansia, Lactobacillus*, and *Enterococcus*. Furthermore, it ameliorated colon tissue damage, as evidenced by the repaired crypt structure and increased goblet cell count (Fig. 3).

As shown in Fig. 3a and b, the Simpson index and Chao index revealed no significant difference in species richness between groups. The Beta diversity of the gut microbiota was assessed by the Bray–Curtis-based PCoA and NMDS. Figure 3c and d shows distinct clustering between the model and intervention groups, implying that SPE treatment altered the gut microbiota composition.

At the phylum level (Fig. 3e), the dominant taxa were *Bacteroidetes, Firmicutes*, and *Actinobacteria*. Compared with the model group, *Bacteroidetes* decreased, while *Actinobacteria* increased in the Inter-group. Histological analysis of colon tissues (Fig. 3f) showed that control mice had intact crypts with orderly epithelial cells and abundant goblet cells. Obese model mice displayed severe crypt distortion and goblet cell loss, while SPE-treated mice exhibited improved crypt organization and reduced epithelial damage.

LEfSe analysis with a logarithmic LDA score threshold of 3 was used to identify bacterial taxa specifically associated with SPE intervention. After the development of obesity in *db/db* mice, the relative abundance of *Proteobacteria, Rikenellaceae, Alcaligenaceae, Alistipes*, and *Corynebacterium* was markedly increased, suggesting that these taxa were promoted under diabetic and inflammatory conditions. Moreover, the genera *Bifidobacterium, Ileibacterium, Erysipelotrichaceae*, and *Desulfovibrionaceae* were predominantly enriched in the Control group, indicating a healthier microbial profile in normal mice. In the Inter group, a high abundance of *Akkermansia, Lactobacillus*, and *Enterococcus* was observed, while the relative abundance of *Bacteroides, Parabacteroides*, and *Oscillibacter* decreased significantly (Fig. 3g and h). These results suggest that SPE supplementation reshaped the gut microbial community structure of *db/db* mice, shifting it toward that of the Control group. Overall, SPE significantly altered gut microbiota composition and helped alleviate obesity-induced colon injury by preserving crypt structure and goblet cells.

**Fig. 4:**
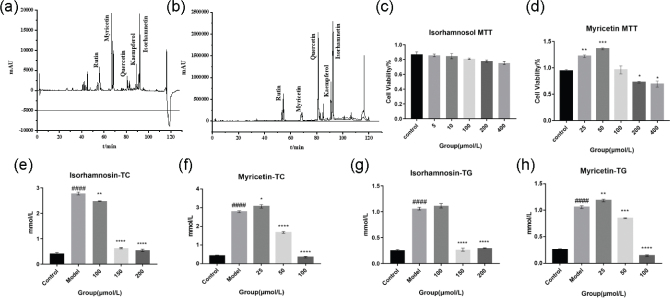
SPE polyphenol analysis and their effects on intracellular cholesterol and triglycerides in HepG2 cells. (a) HPLC analysis of fresh sea buckthorn free phenol. (b) HPLC analysis of SPE freeze-dried powder samples. (c) MTT analysis of HepG2 cells treated with isorhamnetin. (d) MTT analysis of HepG2 cells treated with myricetin. (e) TC levels of HepG2 cells treated with isorhamnetin. (f) TC levels of HepG2 cells treated with myricetin. (g) TG levels of HepG2 cells treated with isorhamnetin. (h) TG levels of HepG2 cells treated with myricetin. Comparison of the Model group (HepG2 cells) with the Control group (blank group) is marked as *, and the comparison of the model group with the Inter-group (HepG2 cells + SPE Polyphenols) is marked as #. * *P* < 0.05, ** *P* < 0.01, *** *P* < 0.001, **** *P* < 0.0001, #### *P* < 0.01. Experiments were performed in triplicate (*n* = 3 independent experiments).

### Effects of major SPE polyphenols isorhamnetin and myricetin on intracellular lipid levels in HepG2 cells

Chromatograms of the fresh sea buckthorn free phenol extract and the SPE freeze-dried powder are shown in Fig. 4a and b. The two profiles displayed highly comparable retention times and peak patterns for the major polyphenols, indicating that the freeze-drying process largely preserved the qualitative composition of SPE. Minor differences in peak intensities likely reflect concentration and matrix effects rather than substantial degradation of individual compounds. After treatment of HepG2 cells with isorhamnetin and myricetin for 24 h, MTT assay results indicated that isorhamnetin had no significant effect on HepG2 cell viability from 5 to 400 μmol/L concentrations. In contrast, myricetin increased cell viability at 25 and 50 μmol/L, showed no significant change at 100 μmol/L compared to control, and decreased cell viability at 200 and 400 μmol/L concentrations (Fig. 4c and d). Therefore, in the following experiments, three concentrations of isorhamnetin (100 150, and 200 μmol/L) and three concentrations of myricetin (25, 50, and 100 μmol/L were selected for further investigation. The results showed that both isorhamnetin and myricetin significantly reduced intracellular TC and TG levels in a concentration-dependent manner (*P* < 0.05). A concentration of 200 μmol/L of isorhamnetin and 100 μmol/L of myricetin can largely reduce cellular TC and TG levels to those comparable with the negative control group (Fig. 4e–h).

### Isorhamnetin primarily targets lipid metabolism in obesity, NAFLD and diabetes

**Fig. 5:**
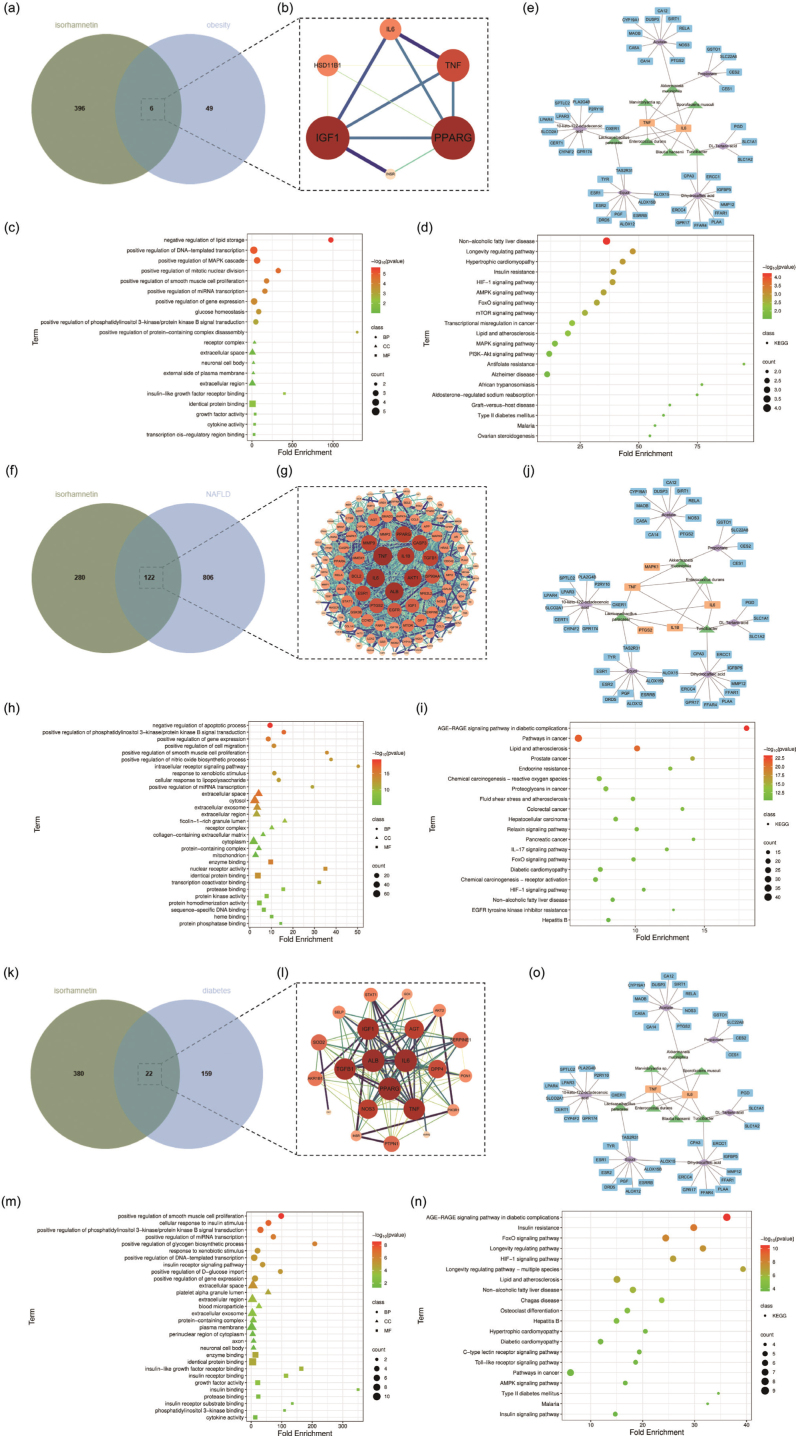
Integrated network pharmacology and microbiota–metabolite–target analysis of isorhamnetin in obesity, NAFLD and diabetes. (a, f, k) Venn diagrams showing the overlaps between predicted isorhamnetin targets and genes associated with obesity, NAFLD, and diabetes, respectively. (b, g, l) PPI networks of the overlapping targets in each disease; node size and color reflect degree values and highlight hub proteins. (c, h, m) GO enrichment bubble plots of the overlapping targets, with fold enrichment on the x-axis, bubble size indicating gene counts, and color representing −log10 (*P*-value). (d, i, n) KEGG pathway enrichment bubble plots, showing that isorhamnetin targets are mainly involved in lipid metabolism–related pathways. (e, j, o) Common target-bacteria-metabolite-target interaction networks for obesity, NAFLD, and diabetes, illustrating the connections among gut bacteria, key metabolites, human targets for metabolites, and isorhamnetin–disease common targets.

For isorhamnetin, intersecting the predicted compound-related targets with disease-associated genes yielded a panel of common targets for obesity, NAFLD and diabetes, respectively (Fig. 5a, f, k). These overlapping genes represent the potential therapeutic targets through which isorhamnetin may modulate metabolic disorders. In the corresponding PPI networks, several lipid- and vascular-related proteins, including PPARG, NOS3, TNF, IL6, IGF1, and TGFB1, occupied central positions with high degree values (Fig. 5d–f), suggesting that isorhamnetin coordinately regulates adipogenic transcription factors, inflammatory mediators, and endothelial function.

GO enrichment analysis revealed that the common targets of isorhamnetin were significantly enriched in biological processes directly related to lipid metabolism, such as lipid catabolic process, cholesterol homeostasis, lipoprotein particle remodeling, and regulation of fatty acid oxidation, as well as in molecular function terms including lipoprotein particle binding and oxidoreductase activity acting on lipid substrates (Fig. 5c, h, m). In agreement with this, KEGG enrichment demonstrated that isorhamnetin-related targets were predominantly clustered in NAFLD, lipid and atherosclerosis, fatty acid degradation, bile secretion and adipocytokine signaling, together with classical insulin signaling and Type II diabetes mellitus pathways (Fig. 5d, e, i).

To further link these targets with the gut–liver axis, a bacteria–metabolite–target interaction network was constructed by integrating the isorhamnetin–disease common targets with differential gut bacteria and fecal metabolites identified in the SPE-treated *db/db* mice. In this network (Fig. 5j, n, o), beneficial genera such as *Enterococcus* and *Akkermansia* were connected to SCFAs, bile acid derivatives, and other lipid-related metabolites, which in turn were linked to core inflammatory and metabolic targets including TNF, IL6, NOS3, and SIRT1. This topology suggests that isorhamnetin-enriched fractions of SPE may enhance lipid catabolism and improve cholesterol/lipoprotein metabolism by reshaping the gut microbiota and its metabolic output, ultimately converging on lipid-metabolism–related targets in obesity, NAFLD, and diabetes.

### Myricetin primarily modulates fatty acid metabolism and adipogenesis

**Fig. 6:**
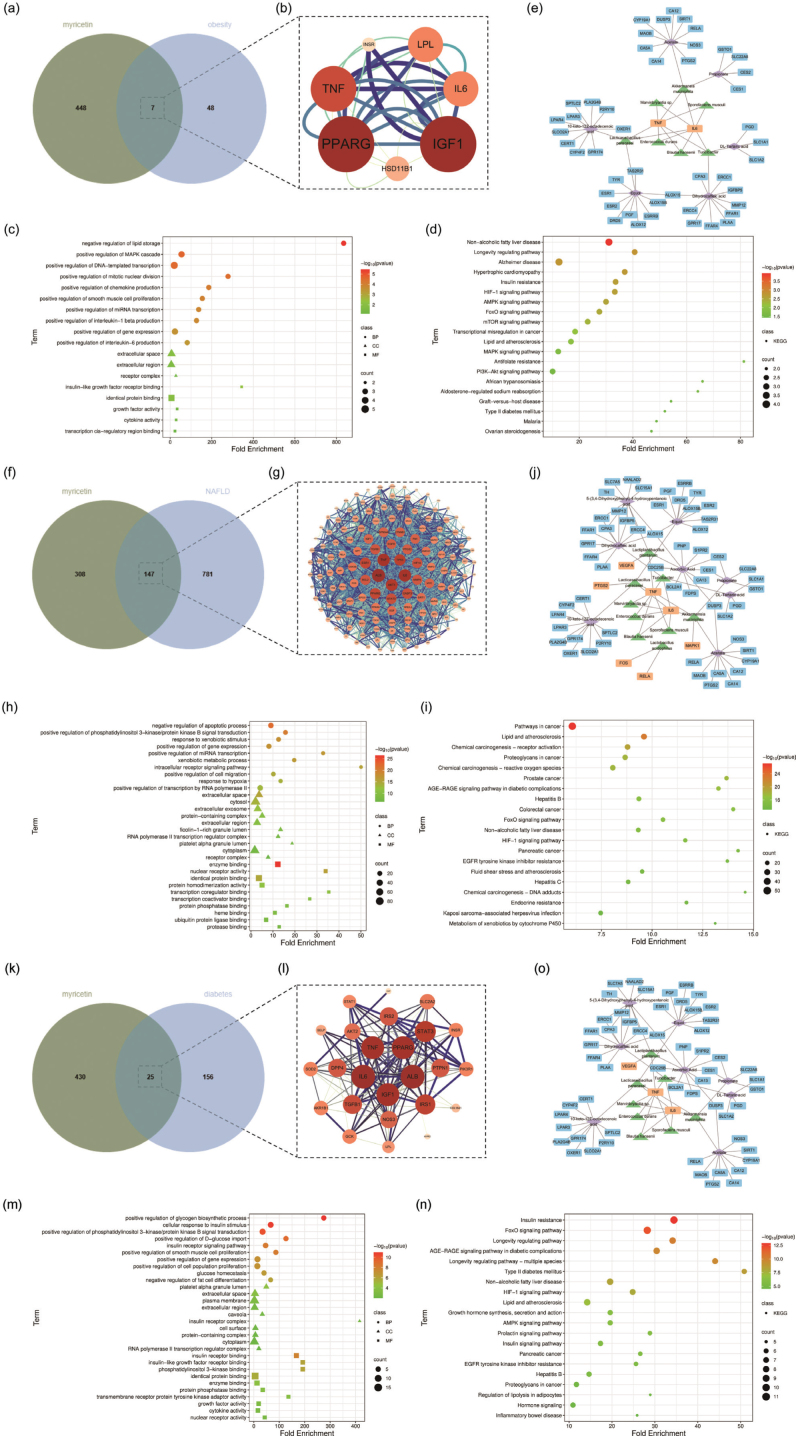
Integrated network pharmacology and microbiota–metabolite–target analysis of myricetin in obesity, NAFLD, and diabetes. (a, f, k) Venn diagrams showing the overlaps between predicted myricetin targets and genes associated with obesity, NAFLD, and diabetes, respectively. (b, g, l) PPI networks of the overlapping targets in each disease; node size and color reflect degree values and highlight hub proteins. (c, h, m) GO enrichment bubble plots of the overlapping targets, with fold enrichment on the x-axis, bubble size indicating gene counts and color representing −log10 (*P*-value). (d, i, n) KEGG pathway enrichment bubble plots, showing that myricetin targets are mainly involved in lipid metabolism–related pathways. (e, j, o) Bacteria–metabolite–target interaction networks for obesity, NAFLD, and diabetes, illustrating the connections among gut bacteria, key metabolites, and myricetin–disease common targets.

A similar network pharmacology strategy was applied to myricetin. Intersecting myricetin-related targets with obesity-, NAFLD-, and diabetes-associated genes identified a set of common targets for each disease (Fig. 6a, f, k). PPI network analysis showed that PPARG, SIRT1, AKT1, TNF, and IL6 were key hub proteins with high connectivity in all three disease networks (Fig. 6b, g, l), indicating that myricetin predominantly acts on adipogenic transcriptional regulation, cellular energy sensing, and inflammatory signaling.

GO enrichment of the myricetin common targets revealed a prominent enrichment in biological processes associated with fatty acid metabolic process, regulation of lipid storage, TG homeostasis, and response to lipid, while cellular component and molecular function annotations pointed to lipid droplets, plasma membrane, extracellular region, lipid binding, and nuclear receptor activity (Fig. 6c, h, m). Consistently, KEGG pathway analysis indicated that myricetin-related targets were significantly enriched in NAFLD, lipid and atherosclerosis, PPAR and AMPK signaling pathways, and insulin resistance and Type II diabetes mellitus (Fig. 6d, e, i).

By further integrating gut microbial and metabolite data, a myricetin-centred bacteria–metabolite–target network was established. In this network (Fig. 6j, n, o), genera that were increased after SPE intervention, particularly *Enterococcus* and *Akkermansia*, were associated with lipid-regulating metabolites (such as SCFAs and bile acid–related molecules), which mapped onto myricetin targets involved in fatty acid synthesis and oxidation, including PPARG, SIRT1, and AKT1. These results indicate that myricetin mainly suppresses de novo lipogenesis, reduces adipocyte hypertrophy, and ectopic lipid accumulation and enhances fatty acid oxidation via the PPAR–AMPK axis and related microbiota–metabolite–host interactions.

Taken together, both isorhamnetin (Fig. 5) and myricetin (Fig. 6) converged on a core set of lipid-metabolism-related targets and pathways across obesity, NAFLD, and diabetes. Isorhamnetin was more prominently enriched in pathways related to lipid catabolism, cholesterol, and lipoprotein remodeling and lipid-driven vascular complications, whereas myricetin showed a stronger association with fatty acid synthesis, adipocyte differentiation and lipid storage. The overlap in hub targets, such as PPARG, TNF, and IL6, together with the consistent enrichment of *Enterococcus* and *Akkermansia* in SPE-treated *db/db* mice, supports a mechanistic model in which sea buckthorn polyphenols ameliorate metabolic disorders by reprogramming host lipid metabolism and reshaping the gut microbiota–metabolite–target axis. This integrated network provides a rational explanation for the observed improvements in dyslipidemia, hepatic steatosis, and glucose–lipid homeostasis in the *db/db* model after SPE intervention.

## Discussion

SPE effectively mitigated obesity, hyperlipidemia, and hepatic steatosis in *db/db* mice. Mechanistically, its protective effects involve multiple interconnected pathways. SPE modulates lipid metabolism by reducing serum and hepatic levels of TG and TC, thereby improving lipid clearance, decreasing hepatic lipid accumulation, and enhancing insulin sensitivity (24). It also exhibits antioxidative and anti-inflammatory effects, decreasing hepatic oxidative stress markers and pro-inflammatory cytokines, thereby alleviating hepatocellular injury (25). Furthermore, SPE modulated the gut–liver axis by modulating intestinal microbiota composition, promoting beneficial bacterial populations, and enhancing SCFA production, which further contributes to improved lipid metabolism and reduced systemic inflammation (24). Complementary in vitro experiments in HepG2 cells demonstrated that isorhamnetin and myricetin, key components of SPE, lowered intracellular TC and TG levels in a concentration-dependent manner without cytotoxicity at optimal doses, supporting the in vivo observations (26). In line with these experimental findings, our network pharmacology analysis showed that the predicted targets of isorhamnetin and myricetin overlapped extensively with obesity-, NAFLD-, and diabetes-related genes and were highly enriched in lipid-metabolism–related processes and pathways, including fatty acid metabolism, cholesterol homeostasis, NAFLD, lipid and atherosclerosis, and PPAR/AMPK signaling. The identification of hub nodes such as PPARG, SIRT1, NOS3, TNF, and IL6 further supports a multitarget, multipathway mode of action by which SPE-derived polyphenols coordinately modulate lipid metabolism and inflammation at the systems level. These findings highlight SPE as a promising therapeutic agent for obesity and related metabolic disorders through coordinated regulation of lipid metabolism, inflammation, and gut microbiota.

Sea buckthorn is rich in polyphenols, which serve as prebiotic substrates capable of selectively promoting the growth of beneficial intestinal bacteria while inhibiting detrimental microbes (27–29). The interaction between polyphenols and gut microbiota is bidirectional: gut microbes metabolize dietary polyphenols into bioactive compounds, including SCFAs, phenolic acids, and flavonoid derivatives, which exert systemic metabolic effects. In *db/db* mice, SPE significantly increased the abundance of beneficial genera such as *Lactobacillus* and *Akkermansia* (30, 31), highlighting its prebiotic activity. *Akkermansia muciniphila* degrades mucin to generate SCFAs, thereby enhancing epithelial barrier integrity, reducing systemic inflammation, and regulating energy homeostasis (16). Meanwhile, *Lactobacillus spp.* can modulate bile acid metabolism via bile salt hydrolase activity, influencing cholesterol solubility and excretion (17). SCFAs, including acetate and propionate, may further activate intestinal gluconeogenesis or hepatic AMPK pathways, collectively contributing to improved lipid profiles such as increased HDL-C levels (18). Moreover, the enrichment of beneficial microbes and increased SCFA production can interact with hepatic metabolism via the gut–liver axis, supporting reductions in hepatic TGs and TC (32). By integrating gut microbiota, fecal metabolite, and host target information, our network pharmacology–based bacteria–metabolite–target network further suggested that genera such as *Akkermansia* and *Enterococcus* are connected to lipid-regulating metabolites and core host targets (e.g. PPARG, TNF, IL6, NOS3), providing a mechanistic link between the prebiotic effects of SPE, SCFA/bile acid signaling, and the downstream modulation of hepatic lipid and inflammatory pathways. Collectively, these findings indicate that modulation of the gut microbiota, particularly through SCFA-mediated signaling and the gut–liver axis, represents a key mechanism underlying the systemic metabolic benefits of SPE.

The major polyphenols in SPE, including isorhamnetin and myricetin, contribute to the regulation of hepatic lipid metabolism by reducing intracellular cholesterol and TG accumulation. Isorhamnetin has been shown to alleviate NAFLD by reducing lipid accumulation in the liver, improving levels of TGs and cholesterol, and enhancing liver function indices (33). Myricetin supplementation has been reported to improve glucose metabolism and lipid profiles in mouse models, suggesting its potential as a therapeutic agent for managing type 2 diabetes mellitus (34). These polyphenols mechanistically modulate key metabolic pathways, including the inhibition of de novo lipogenesis and the enhancement of fatty acid *β*-oxidation, potentially through AMPK activation. Polyphenols have been found to stimulate AMPK, lower lipids, and inhibit accelerated atherosclerosis in diabetic LDL receptor-deficient mice, indicating their role in lipid metabolism regulation (35). Furthermore, polyphenol-mediated AMPK activation results in lipogenesis inhibition and lipophagy, contributing to their lipid-lowering effects (36). Consistent with these reports, our network pharmacology analysis revealed that isorhamnetin- and myricetin-related targets are significantly enriched in NAFLD, lipid and atherosclerosis, fatty acid degradation, PPAR and AMPK signaling pathways, with overlapping hub genes involved in both lipid metabolism and inflammatory control. This systems-level evidence indicates that the direct hepatocellular actions of isorhamnetin and myricetin observed in HepG2 cells are embedded in a broader regulatory network that integrates lipid synthesis, oxidation, cholesterol transport, and inflammatory responses, complementing the in vivo effects of SPE in *db/db* mice. These findings support the notion that isorhamnetin and myricetin contribute to hepatic cholesterol homeostasis by directly attenuating lipid accumulation, complementing our systemic effects observed in vivo in *db/db* mice treated with SPE. Moreover, the regulation of intracellular cholesterol and TGs levels by these polyphenols could synergize with gut microbiota-mediated effects, such as SCFA production and bile acid metabolism, highlighting a coordinated network of hepatic and intestinal mechanisms through which SPE exerts its protective metabolic effects (37).

The gut–liver axis appears to be central to the systemic metabolic effects of SPE, as modulation of gut microbiota, enhancement of SCFA production, and regulation of bile acid metabolism likely interact with hepatic lipid and glucose homeostasis. These interactions may collectively contribute to improved serum lipid profiles, reduced hepatic TG and cholesterol levels, and alleviation of metabolic inflammation. However, several limitations exist in the current study. First, the potential synergistic effects of multiple polyphenols present in SPE were not fully explored, leaving uncertainties regarding whether combined actions of these compounds may produce additive or synergistic benefits. Second, the study did not include long-term pharmacokinetic or bioavailability assessments, which are critical to understanding how SPE compounds are absorbed, metabolized, and maintained at effective concentrations in vivo. Additionally, the study used an animal model and in vitro cell lines, and translational relevance to humans remains to be validated. From the perspective of network pharmacology, another limitation is that the present analysis primarily relied on metabolite measurements and in silico network predictions and did not include protein-level or signaling activity assays, which limits the ability to fully confirm functional changes in the implicated pathways; thus, the predicted compound–target interactions and network hubs require further validation by proteomic, phosphoproteomic, or targeted functional studies. Future investigations integrating proteomic analyses, polyphenol interaction studies, and pharmacokinetic profiling are necessary to comprehensively elucidate the mechanisms underlying SPE’s systemic metabolic benefits and to optimize its therapeutic potential. Although TNF, IL6, PPARG, and AKT1 emerged as key hubs in the network pharmacology analysis, we did not quantify circulating inflammatory cytokines in this study due to limited serum volume. Future work should measure serum TNF-*α*, IL-6, and related markers to experimentally validate the predicted inflammatory targets and further link the in-silico network to systemic inflammation.

## Conclusion

SPE improves lipid metabolism in *db/db* mice primarily through the coordinated regulation of gut microbiota and hepatic cholesterol homeostasis pathways. SPE administration enriched beneficial intestinal microbes such as *Lactobacillus* and *Akkermansia*, thereby enhancing the gut–liver axis and contributing to reductions in serum and hepatic TG and TC levels. Major polyphenols in SPE, including isorhamnetin and myricetin, regulated intracellular lipid accumulation in hepatocytes. Network pharmacology analysis further revealed that the predicted targets of these polyphenols overlapped extensively with obesity-, NAFLD-, and diabetes-related genes, and were mainly enriched in lipid-metabolism–related processes and pathways, such as fatty acid metabolism, cholesterol homeostasis, NAFLD, lipid and atherosclerosis, and PPAR/AMPK signaling. Integration of gut microbiota, fecal metabolite, and host target information suggested a gut microbiota–metabolite–host target axis involving beneficial taxa (e.g. *Akkermansia, Enterococcus*) and key hubs including PPARG, SIRT1, TNF, and IL6. The dual actions on gut microbiota and liver metabolism, together with the multitarget and multipathway regulatory networks identified in silico, underscore a synergistic mechanism by which SPE alleviates dyslipidemia and reduces metabolic stress. These findings provide a mechanistic foundation supporting SPE as a promising functional food or nutraceutical candidate for the prevention and management of dyslipidemia and related metabolic disorders. Future studies are necessary to explore long-term efficacy, polyphenol bioavailability, synergistic interactions, and translational potential in humans, as well as to experimentally validate the key targets and pathways predicted by network pharmacology.

## Supplementary Material



## References

[1] Segula D. Complications of obesity in adults: a short review of the literature. Malawi Med J 2014; 26(1): 20–4. Available from: https://www.ncbi.nlm.nih.gov/pmc/articles/PMC4062780/ [cited 21 July 2025]. 24959321 PMC4062780

[2] Després JP.Obesity and lipid metabolism: relevance of body fat distribution. Curr Opin Lipidol 1991; 2(1): 5. doi: 10.1097/00041433-199102000-00003

[3] Benfante R, Yano K, Hwang LJ, Curb JD, Kagan A, Ross W. Elevated serum cholesterol is a risk factor for both coronary heart disease and thromboembolic stroke in Hawaiian Japanese men. Implications of shared risk. Stroke 1994; 25(4): 814–20. doi: 10.1161/01.STR.25.4.814 8160226

[4] Rodriguez BL, D’Agostino R, Abbott RD, Kagan A, Burchfiel CM, Yano K, et al. Risk of hospitalized stroke in men enrolled in the Honolulu Heart Program and the Framingham Study. Stroke 2002; 33(1): 230–6. doi: 10.1161/hs0102.101081 11779915

[5] Eckel RH, Alberti K, Grundy SM, Zimmet PZ. The metabolic syndrome. Lancet 2010; 375(9710): 181–3. doi: 10.1016/S0140-6736(09)61794-3 20109902

[6] Luo Y, Wang L, Lv Y, Wu X, Hou C, Li J. Regulation mechanism of silkworm pupa oil PUFAs on cholesterol metabolism in hepatic cell L-02. J Sci Food Agric 2020; 100(4): 1418–25. doi: 10.1002/jsfa.10115 31667852

[7] Niu Y, Hu X, Song Y, Wang C, Luo P, Ni S, et al. Blautia coccoides is a newly identified bacterium increased by leucine deprivation and has a novel function in improving metabolic disorders. Adv Sci (Weinh) 2024; 11(18): 2309255. doi: 10.1002/advs.202309255 38429906 PMC11095201

[8] Suryakumar G, Gupta A. Medicinal and therapeutic potential of Sea buckthorn (*Hippophae rhamnoides* L.). J Ethnopharmacol 2011; 138(2): 268–78. doi: 10.1016/j.jep.2011.09.024 21963559

[9] Fu Y, Liu Z, Wang K, Li X, Fu J, Tan Y, et al. Fermented sea buckthorn compound juice inhibits colorectal cancer growth by regulating immunity and the gut microbiome. J Funct Foods 2024; 121: 106408. doi: 10.1016/j.jff.2024.106408

[10] Chen A, Feng X, Dorjsuren B, Chimedtseren C, Damda TA, Zhang C. Traditional food, modern food and nutritional value of Sea buckthorn (*Hippophae rhamnoides* L.): a review. J Future Foods 2023; 3(3): 191–205. doi: 10.1016/j.jfutfo.2023.02.001

[11] Li Y, Chen Z, Wang J, Wang T, Liu W, Wu H, et al. Recent advances in innovative extraction techniques, comprehensive composition analysis, bioactivity assessment, and development of efficient delivery systems for sea buckthorn oil. Trends Food Sci Technol 2025; 159: 104944. doi: 10.1016/j.tifs.2025.104944

[12] Rendine M, Marino M, Martini D, Riso P, Møller P, Del Bo’ C. Effect of (Poly)phenols on lipid and glucose metabolisms in 3T3-L1 adipocytes: an integrated analysis of mechanistic approaches. Curr Obes Rep 2025; 14(1): 64. doi: 10.1007/s13679-025-00656-6 40767889 PMC12328513

[13] Huang H, Li Y, Gui F, Yang P, Zhang J, Li W, et al. Optimizing the purification process of polyphenols of sea buckthorn seed and its potential freshness effect. LWT 2023; 173: 114380. doi: 10.1016/j.lwt.2022.114380

[14] Wu B, Bhatnagar R, Indukuri VV, Chopra S, March K, Cordero N, et al. Intestinal mucosal barrier function restoration in mice by maize diet containing enriched Flavan-4-Ols. Nutrients 2020; 12(4): 896. doi: 10.3390/nu12040896 32218287 PMC7230161

[15] He M, Shi B. Gut microbiota as a potential target of metabolic syndrome: the role of probiotics and prebiotics. Cell Biosci 2017; 7: 54. doi: 10.1186/s13578-017-0183-1 29090088 PMC5655955

[16] Plovier H, Everard A, Druart C, Depommier C, Van Hul M, Geurts L, et al. A purified membrane protein from Akkermansia muciniphila or the pasteurized bacterium improves metabolism in obese and diabetic mice. Nat Med 2017; 23(1): 107–13. doi: 10.1038/nm.4236 27892954

[17] Joyce SA, MacSharry J, Casey PG, Kinsella M, Murphy EF, Shanahan F, et al. Regulation of host weight gain and lipid metabolism by bacterial bile acid modification in the gut. Proc Natl Acad Sci USA 2014; 111(20): 7421–6. doi: 10.1073/pnas.1323599111 24799697 PMC4034235

[18] den Besten G, van Eunen K, Groen AK, Venema K, Reijngoud DJ, Bakker BM. The role of short-chain fatty acids in the interplay between diet, gut microbiota, and host energy metabolism. J Lipid Res 2013; 54(9): 2325–40. doi: 10.1194/jlr.R036012 23821742 PMC3735932

[19] Hopkins AL. Network pharmacology: the next paradigm in drug discovery. Nat Chem Biol 2008; 4(11): 682–90. doi: 10.1038/nchembio.118 18936753

[20] Traditional Chinese medicine network pharmacology: theory, methodology and application. Chin J Nat Med 2013; 11(2): 110–20. doi: 10.3724/SP.J.1009.2013.00110 23787177

[21] Chen G, Gou B, Du Y, Zhou Z, Bai Y, Yang Y, et al. Predicting the molecular mechanism of ginger targeting PRMT1/BTG2 axis to inhibit gastric cancer based on WGCNA and machine algorithms. Phytomedicine 2025; 143: 156892. doi: 10.1016/j.phymed.2025.156892 40446574

[22] Dai Z, Wang H, Shen Q, Hu Y, Xue Y. Raw and heat-treated quinoa protein protects against glucose metabolism disorders in high-fat diet (HFD)-induced mice by reshaping gut microbiota and fecal metabolic profiles. Food Funct 2024; 15(18): 9409–19. doi: 10.1039/D4FO02904F 39189421

[23] Zhao L, Wen E, Upur H, Tian S. High performance liquid chromatography-diode array detector method for the simultaneous determination of five compounds in the pulp and seed of sea buckthorn. Pharmacogn Mag 2017; 13(49): 136–40. doi: 10.4103/0973-1296.197656 28216897 PMC5307898

[24] Wang Z, Zhou S, Jiang Y. Sea buckthorn pulp and seed oils ameliorate lipid metabolism disorders and modulate gut microbiota in C57BL/6J mice on high-fat diet. Front Nutr 2022; 9: 1067813. doi: 10.3389/fnut.2022.1067813 36570130 PMC9773879

[25] Wang Z, Zhao F, Wei P, Chai X, Hou G, Meng Q. Phytochemistry, health benefits, and food applications of sea buckthorn (Hippophae rhamnoides L.): a comprehensive review. Front Nutr 2022; 9: 1036295. doi: 10.3389/fnut.2022.1036295 36562043 PMC9763470

[26] Kim DY, Kim SR, Jung UJ. Myricitrin ameliorates hyperglycemia, glucose intolerance, hepatic steatosis, and inflammation in high-fat diet/streptozotocin-induced diabetic mice. Int J Mol Sci 2020; 21(5): 1870. doi: 10.3390/ijms21051870 32182914 PMC7084451

[27] Plamada D, Vodnar DC. Polyphenols–gut microbiota interrelationship: a transition to a new generation of prebiotics. Nutrients 2022; 14(1): 137. doi: 10.3390/nu14010137 PMC8747136 35011012

[28] Wang K, Xu Z, Liao X. Bioactive compounds, health benefits and functional food products of sea buckthorn: a review. Crit Rev Food Sci Nutr 2022; 62(24): 6761–82. doi: 10.1080/10408398.2021.1905605 33783272

[29] Li Y, Rahman SU, Huang Y, Zhang Y, Ming P, Zhu L, et al. Green tea polyphenols decrease weight gain, ameliorate alteration of gut microbiota, and mitigate intestinal inflammation in canines with high-fat-diet-induced obesity. J Nutr Biochem 2020; 78: 108324. doi: 10.1016/j.jnutbio.2019.108324 32004926

[30] Nogal A, Tettamanzi F, Dong Q, Louca P, Visconti A, Christiansen C, et al. A fecal metabolite signature of impaired fasting glucose: results from two independent population-based cohorts. Diabetes 2023; 72(12): 1870–80. doi: 10.2337/db23-0170 37699401 PMC10658071

[31] Wu T, Chu X, Cheng Y, Tang S, Zogona D, Pan S, et al. Modulation of gut microbiota by Lactobacillus casei fermented raspberry juice in vitro and in vivo. Foods 2021; 10(12): 3055. doi: 10.3390/foods10123055 34945605 PMC8702086

[32] Singh S, Kriti M, Catanzaro R, Marotta F, Malvi M, Jain A, et al. Deciphering the gut–liver axis: a comprehensive scientific review of non-alcoholic fatty liver disease. Livers 2024; 4(3): 435–54. doi: 10.3390/livers4030032

[33] Ganbold M, Owada Y, Ozawa Y, Shimamoto Y, Ferdousi F, Tominaga K, et al. Isorhamnetin alleviates steatosis and fibrosis in mice with nonalcoholic steatohepatitis. Sci Rep 2019; 9: 16210. doi: 10.1038/s41598-019-52736-y 31700054 PMC6838085

[34] Babotă M, Frumuzachi O, Tanase C, Mocan A. Efficacy of myricetin supplementation on glucose and lipid metabolism: a systematic review and meta-analysis of in vivo mice studies. Nutrients 2024; 16(21): 3730. doi: 10.3390/nu16213730 39519561 PMC11547919

[35] Zang M, Xu S, Maitland-Toolan KA, Zuccollo A, Hou X, Jiang B, et al. Polyphenols stimulate AMP-activated protein kinase, lower lipids, and inhibit accelerated atherosclerosis in diabetic LDL receptor–deficient mice. Diabetes 2006; 55(8): 2180–91. doi: 10.2337/db05-1188 16873680

[36] Mirahmadi SMS, Hoseini S, Mirahmadi SMS. Therapeutic properties of polyphenols affect AMPK molecular pathway in hyperlipidemia. Preprints; 2023. Available from: https://www.preprints.org/manuscript/202301.0528/v1 [cited 10 October 2025].

[37] He YJ, You CG. The potential role of gut microbiota in the prevention and treatment of lipid metabolism disorders. Int J Endocrinol 2020; 2020: 8601796. Available from: https://pmc.ncbi.nlm.nih.gov/articles/PMC7509545/ [cited 10 October 2025]. 33005189 10.1155/2020/8601796 PMC7509545

